# Polymorphism of the FABP2 gene: a population frequency analysis and an association study with cardiovascular risk markers in Argentina

**DOI:** 10.1186/1471-2350-8-39

**Published:** 2007-06-26

**Authors:** Laura C Gomez, Sebastián M Real, Marta S Ojeda, Sergio Gimenez, Luis S Mayorga, María Roqué

**Affiliations:** 1 Laboratory of Cellular and Molecular Biology, Faculty of Medical Sciences, National University of Cuyo, Mendoza CP:5500, Argentina; 2 Faculty of Chemistry, Biochemistry and Pharmacy, National University of San Luis, San Luis, Argentina; 3 Sanatorio Fleming-OSEP, Mendoza, Argentina

## Abstract

**Background:**

The FABP2 gene encodes for the intestinal FABP (IFABP) protein, which is expressed only in intestinal enterocytes. A polymorphism at codon 54 in exon 2 of the FABP2 gene exchanges an Alanine (Ala), in the small helical region of the protein, for Threonine (Thr). Given the potential physiological role of the Ala54Thr FABP2 polymorphism, we assess in this study the local population frequency and analyze possible associations with five selected markers, i.e. glycemia, total cholesterol, body mass index (BMI), hypertension, and high Cardiovascular Risk Index (CVR index).

**Methods:**

We studied 86 men and 116 women. DNA was extracted from a blood drop for genotype analysis. Allele frequencies were calculated by direct counting. Hardy Weinberg Equilibrium was evaluated using a Chi-square goodness of fit test.

For the polymorphism association analysis, five markers were selected, i.e. blood pressure, Framingham Risk Index, total cholesterol, BMI, and glycemia.

For each marker, the Odds Ratio (OR) was calculated by an online statistic tool.

**Results:**

Our results reveal a similar population polymorphism frequency as in previous European studies, with **q = 0.277 **(95% confidence limits 0.234–0.323). No significant association was found with any of the tested markers in the context of our Argentine nutritional and cultural habits. We did, however, observe a tendency for increased Cholesterol and high BMI in Thr54 carriers.

**Conclusion:**

This is the first study to look at the population frequency of the Thr54 allele in Argentina. The obtained result does not differ from previously reported frequencies in European populations. Moreover, we found no association between the Thr54 allele and any of the five selected markers. The observed tendency to increased total cholesterol and elevated BMI in Thr54 carriers, even though not significant for p < 0.1 could be worth of further investigation to establish whether the Thr54 variant should be taken into consideration in cardiovascular prevention strategies.

## Background

Fatty acid-binding proteins (FABPs) are small intracellular polypeptides found in many tissues, involved in fatty acid transfer and metabolism [1] and encoded by a family of different genes. The FABP2 gene encodes intestinal FABP protein (IFABP), and is only expressed in the absorptive simple columnar epithelial cells of the intestine (enterocytes) [2]. Several functions of IFABP protein have been proposed, these include the facilitation of cellular uptake and/or transport of long-chain fatty acids within enterocytes [3]. The IFABP protein contains two beta-sheet structures surround a cavity into which the ligand binds [4]. The major conformational adjustment between the structure of free IFABP and IFABP bound to fatty acid, occurs at a tight turn containing residues 54 and 55. These residues shift position when long-chain fatty acids are bound to the protein. Therefore, even a subtle change in the amino acid sequence of this turn could affect the structural properties of IFABP in such a way as to alter its ligand affinity [5].

A polymorphism at codon 54 in exon 2 of the human FABP2 gene exchanges an Alanine (Ala) in the small helical region of the protein for Threonine (Thr). Different studies suggest that the Ala-to-Thr substitution is in fact a functional mutation [6,7]. It is reasonable to speculate that if the FABP2 gene polymorphism in any way modifies the absorption of fatty acids, it could in turn affect the lipid metabolism and/or correlate with cardiovascular disease (CVD) risk. Earlier studies have shown that the IFABP Thr54 allele is significantly associated with higher total cholesterol, with stroke incidence [8], elevation of fasting and postprandial triglyceride [9], insulin resistance [2,6], and higher nonesterified fatty acid (NEFA) concentrations [10]. There are many contradicting studies, however, that have found non-significant association with these parameters [11-14]. In the light of the potential physiological role of the FABP2 polymorphism, we assessed the local population frequency of the Thr54 allele and analyzed its possible associations with five selected markers, i.e. glycemia, total cholesterol, body mass index (BMI), hypertension and cardiovascular risk index (CVR index). Our results reveal a similar population polymorphism frequency as in previous European studies and a not significant association with none of the tested markers in the context of our argentine nutritional and cultural habits.

## Methods

### Subjects

We recruited 202 volunteers (mean age 53 years) from various Public Administration Offices, including 86 men (aged between 35 and 75 years, mean age 51) and 116 women (aged between 45 and 75 years, mean age 56). The means in the sample were: cholesterol level 198,34 (190,52 for men and 204,27 for women), diastolic blood pressure: 79,38 (82,93 for men and 76,96 for women), systolic blood pressure: 127,22 (131,15 for men and 124,26 for women) and BMI: 28,45 (28,86 for men and 28,13 for woman). In the 202 subjects, there were only 11 diabetics. On receiving written informed consent from all participants included in this population screening study, a drop of blood was collected for total cholesterol and glycemia analysis using commercial kits (Accu-Chek Instant Plus, Roche Diagnostic). A second drop of blood was collected on a DNAase free paper (Nucleic Paper, Biodynamics S.R.L, Buenos Aires, Argentina) for DNA extraction. Systolic and diastolic blood pressure was measured by a physician. BMI was calculated as weight (kg) divided by height squared (m^2^). The ethical committee of the Public Health Care Program for Government Employees (OSEP, Obra Social del Empleado Publico) approved the study.

### Cardiovascular risk assessment by calculation of the Framingham Risk Index

The cardiovascular risk was calculated for each individual by the Framingham Index according to the National Cholesterol Education Program (NCEP) Adult Treatment Panel (ATP) III guidelines [15]. This index takes into account the following variables: sex, age, blood pressure, total cholesterol, HDL and smoking status. The HDL measure was excluded from this study because of its low impact on the total score for the risk calculation. The total score was obtained by summing the partial scores for each of the variables mentioned. The total score of 10 was established as the threshold, with a 0 to 9 total score implying *low risk status *and a 10 or higher total score implying *high risk status*.

### Genotype analysis

To extract DNA, a piece of 2 mm radio was cut out from the blood drop on DNAse free paper, added to 100 μl sterile water, and then incubated for 30 minutes at 95°C. For PCR reaction 10 μl were used. Some DNA samples which did not amplify well were diluted 1:10 or 1:20.

DNA was amplified in a total volume of 20 μl, with the upstream primer: 5'ACAGGTGTTAATATAGTGAAAAG3' and the downstream primer: 5' TACCCTGAGTTCAGTTCCGTC3' [6]. The PCR program consisted of an initial and final hold of 94°C and 72°C for 3 minutes, and 30 cycles of 94°C, 55°C, and 72°C, with each step of 30 seconds duration.

For Restriction Fragment length Polymorphism (RFLP) analysis, 5 μl of PCR product were incubated with 0.4 μl of enzyme CfoI (GCG/C) (10 U/μl, Promega) in a final volume of 10 μl for 1 hour at 37°C.

The products were run on a 10% non-denaturing PAGE for 50 minutes at 110 V. Bands were observed after Ethidium bromide staining and UV light exposure.

### Allele frequency and polymorphism association analysis

Allele frequencies were calculated by direct counting. Hardy Weinberg Equilibrium was evaluated using a Chi-square goodness of fit test.

For the polymorphism association analysis, five markers were selected, i.e. blood pressure, Framingham Risk Index, total cholesterol, BMI, and glycemia. Each parameter was assigned a threshold value, established by the RCP Program, as follows:

1-Hypertension >140 mmHg systolic or >90 mmHg diastolic; 2-Framingham Risk Index >9 total score; 3-BMI >24 kg/m2; 4-Total cholesterol > 240 mg/dl; 5-Glycemia >110 mg/dl after 8 h fasting or >140 mg/dl postprandial.

For each marker, the Odds Ratio (OR) was calculated by the online statistic tool [16].

## Results

### The Thr54 allele frequency in Argentina

To assess the polymorphism frequency in our region, DNA samples of 202 individuals (86 men and 116 women) were analyzed for the Ala/Thr polymorphism. The included subjects belonged to an ongoing Regional Cardiovascular Prevention Program (RCP program), organised by the Public Health Care Program for Government Employees (OSEP) of Mendoza (Argentina). This programme focuses on the assessment of an individual cardiovascular risk index in order to apply prevention strategies in the analyzed population.

The observed population genotype composition was: 105 Ala/Ala homozygous, 82 Ala/Thr heterozygous and 15 Thr/Thr homozygous (Table 1).

**Table 1:** Genotype analysis in the study subjects

	**Homozygous Ala54/Ala54**	**Homozygous Ala54/Thr54**	**Homozygous Thr54/Thr54**
**Observed number**	105	82	15
**Expected number**	105.5	81	15.5

**X**^2^**n.s. p > 0.05 q(Thr54)= 0.2777 (95% CI: 0.234–0.323) Sample size: 202**			

In a sample of 202 individuals, genotype analysis was performed for the polymorphism Ala/Thr. The observed number of homozygous and heterozygous was not significantly different from that expected in a population in Hardy Weinberg Equilibrium. The Thr54 frequency found in the Argentine populations was q = 0.277.

Eventhough the province of Mendoza has a total population of almost 1.600.000 individuals, it presents a non-homogenous population distribution provoked by the migration of families to the surroundings of the capital city of Mendoza. We therefore decided to carefully analyze our genotype composition results in order to detect whether our sample could be affected by endogamic behaviour. The cluster of inhabitants around the city could lead to inbreeding and consequently reduce the number of observed homozygous, and affect the allele frequency. In order to assess whether this was interfering with our population sample, the amount of observed heterozygous and homozygous for the Ala54Thr polymorphism was used in the formula F = He-Ho/He (where He and Ho are expected and observed heterozygous). However, a negative **(-) 0,02 **inbreeding rate was found, confirming there is no endogamic behaviour in our population that could have affected the allele frequency.

The calculated Thr54 allele frequency was **q = 0.277 **(95% confidence limits 0.234–0.323). In a subsequent analysis of the equilibrium of the populations genotypes, the results revealed that the proportion of hetero and homozygous did not differ significantly from the expected genotypes for a population in Hardy Weinberg equilibrium (Table 1) (p > 0.05, Chi squared goodness of fit), which is consistent with our first finding of non-endogamic behaviour.

The assessment of the polymorphism frequency in Argentina with similar values as in European countries [17] encouraged us to subsequently analyze possible genotype-phenotype associations.

### Polymorphism association analysis reveals no significant impact on CVD risk markers

For the genotype-phenotype association assessment, the phenotypic information of the 202 individuals included in the study was used. Association analysis with allele Thr54 was performed on five selected parameters, i.e. glycemia, total cholesterol, BMI, hypertension, and cardiovascular risk (calculated by the Framingham Risk Index). Comparisons were performed between subjects that carried at least one Thr54 allele (homozygous Thr/Thr and heterozygous Ala5Thr) and non-carriers of the allele Thr54 (homozygous Ala/Ala). For each parameter, a threshold value was established by the RCP Program (see Methods).

Our study reveals **no significant association **(p > *0.05) *between the Thr54 allele and any of the analyzed parameters (Table 2). The ORs represent the magnitude of association between the genotype and disease, consequently ORs with a 95%CI including the value 1 are indicative of *no difference *between Thr54 carriers and non-carriers for that factor. We analysed the five markers in male Thr54 carriers respect to female Thr54 carriers. We did not find any significant difference (for p = 0,05) between gender for BMI, cholesterol, blood pressure, and glycemia.

**Table 2:** Association analysis of the Thr54 polymorphism and phenotypic parameters

**Phenotypic parameters and threshold values**	**OR**	**CI (p = 0.05)**	**Significance**
**Cholesterol > 240 mg/ml**	2.01	0.87–4.65	NS
**BMI>24 kg/m2**	1.44	0.7–2.98	NS
**Cardiovascular risk >9**	1.25	0.62–2.5	NS
**Diastolic blood pressure > 90 mmHg**	0.93	0.51–1.69	NS
**Systolic blood pressure > 140 mmHg**	0.86	0.44–1.47	NS
**Glycemia > 140 mg/dl postprandial**	0.76	0.33–1.76	NS

ORs were calculated as ratio between polymorphism carriers and non-carriers with high blood pressure (>140 mmHg systolic or 90 mmHg diastolic), high Framingham Risk Index (>9) high cholesterol (> 240 mg/dl), high BMI (>24 kg/m2), and high glycemia (>110 mg/dl after 8 h fasting or >140 postprandial mg/dl). 95% Confidence Intervals for p = 0.05 are shown. All CI include OR = 1, indicating no significant association between the polymorphism and the parameters tested.

These results are in accordance with other previous publications that found no major contribution of allele Thr54 on these phenotypic parameters [11-14].

**Figure 1:**
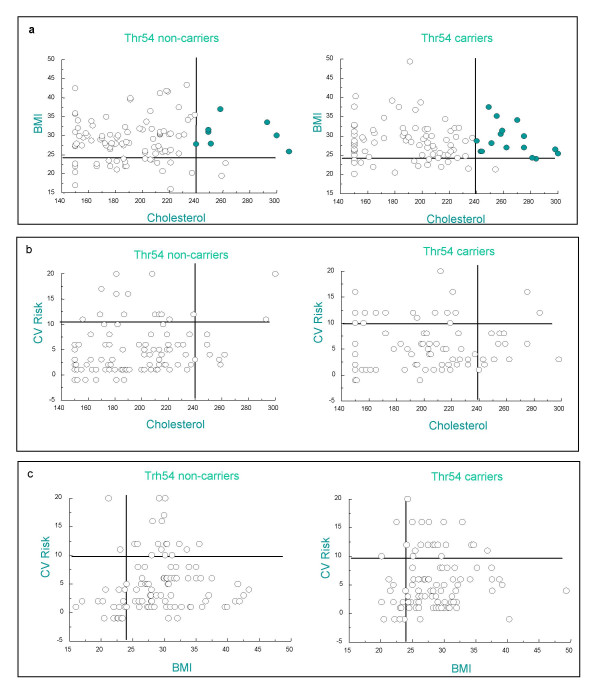
**Association of Thr54 carriers with the combination of markers: i.e. cholesterol, BMI and cardiovascular risk index**. Sample distribution for Thr54 carriers (homozygous Thr/Thr and heterozygous Ala/Thr) and non-carriers (homozygous Ala/Ala) regarding two features: (a) BMI and Choslesterol (b) Cardiovascular risk index (CV Risk) and Cholesterol. (c) Cardiovascular risk index (CV Risk) and BMI. The crossing lines show the threshold values for BMI (24 kg/m2), cholesterol (220 mg/dl), and high risk (9 total score)

Despite the non-significant association results, we consider it worth noting that the ORs for Cholesterol (2.01), BMI (1.44) and CVD risk (1.25) are >1. When we analyzed the distribution of Thr54 carriers and non-carriers combining two of these three parameters, we observed an increased number of polymorphism carriers with high cholesterol and high BMI (Figure 1). Notice in Figure 1a that the quadrant corresponding to high cholesterol *and *high BMI shows more carrier individuals (17/97) than non-carriers (10/105). This not significant difference for p < 0.1 but subtle tendency was not observed in the rest of the parameter combinations that were analyzed (data not shown). These results reveal that even though the markers for Cholesterol and BMI do not show a significant association with the polymorphism when they are analyzed on their own, a subtle tendency is observed when they are analyzed in combination.

## Discussion

There are no previous reports about the prevalence of the Thr54 FABP2 variant in our region. In the sample tested, the observed Thr54 frequency (q = 0,277) is similar to that reported in eleven different European countries (0.276) [17], suggesting that the colonising European populations, mainly from Italy and Spain, introduced the same original allele frequency in this continent and that this frequency has remained conserved. The fact that the proportion of carriers has not changed and that the polymorphism is in a Hardy Weinberg equilibrium suggests that there is no significant natural selection pressure acting against individuals with the Thr54 FABP2 variant living in Mendoza, Argentina. Other South American frequency analysis of Thr54 were reported by a Chilean group, who found a q = 0.4 in 63 obese and not obese women [18], and a Brazilian study that reported a q = 0.25 in 1042 diabetes type 2 individuals [19]. Even though both analyses are performed on specific study sample, i.e. obese and diabetic individuals, the Brazilian frequency in 1042 individuals is similar to that observed by our group in Argentina.

It is well known that the single nucleotide polymorphism (SNP) at codon 54 is a missence variant that has a definite effect on the primary structure of the protein and affects its fatty acid binding properties. It is not known, however, whether this change can affect the lipid metabolism of carriers [20]. Many earlier studies have reported associations between this polymorphism and insulin resistance, BMI, dyslipidemia, stroke, metabolic syndromes and hypertriglyceridemia [2,6,8-10,21-23]. In contrast, other studies report no association with the same parameters [11-14]. Part of the discrepancy among the studies may come from the different dietary habits of the analyzed populations. For example, the lack of association found in a Finnish population [24] could be related to its prevailing fish diet where the Thr54 variant may not cause any phenotypical manifestation. Therefore, it was important to assess some of these associations in Argentina, characterized by the consumption of significant amounts of beef (i.e. 68 kg of beef/year/habitant). This diet is rich in proteins and fats and poor in fibres and fish [25]. In addition, it was relevant for the local RCP Program to develop health prevention strategies based on educational programs of life style and nutritional habits.

Our results indicate that in the assessed population, the allele Thr54 does not influence the glycemia levels nor the blood pressure (odds ratio approaching 1).

The Cholesterol levels, BMI and CVR index are not significantly influenced by the Thr54, but show ORs >1. These tendencies for increased risk are better manifested when BMI and Cholesterol are analysed in a combination. This observation does, however, not prove association or causality. The sample size was small, and these observed tendencies require further investigation in order to reveal whether the influences become significant and whether it could be related to the meat and fat-rich diet prevailing in Argentina.

## Conclusion

We present the first study investigating the Thr54 allele frequency in Argentina and its association with five cardiovascular risk markers. The population frequency of the Thr54 allele in Argentina does not differ from previously reported frequencies in European populations. Moreover, we found no association between the Thr54 allele and any of the five selected markers. Although the observed tendency for increased total cholesterol and elevated BMI in Thr54 carriers was not significant, this will require further investigation to establish whether the Thr54 variant should be taken into consideration in cardiovascular prevention strategies in our country.

## Competing interests

The author(s) declare that they have no competing interests.

## Authors' contributions

LCG performed the DNA sampling, the molecular assays, the frequency analysis, the phenotypic association studies and the revision of the manuscript. SMR contributed to the DNA sampling and the genotyping experiments. MSO provided the original idea and the PCR primers set. SG is the head of the RCP Program. LSM advised on the design of the study, on the experimental work, and on the critical revision of the manuscript. MR developed and guided the project and drafted the manuscript. All authors read and approved the final manuscript.

## Pre-publication history

The pre-publication history for this paper can be accessed here:


